# Use of Mohr diagrams to predict fracturing in rock masses, with applications for predicting sub-surface behavior

**DOI:** 10.1016/j.isci.2024.110272

**Published:** 2024-06-14

**Authors:** D.C.P. Peacock, David J. Sanderson, Bernd Leiss

**Affiliations:** 1 Geoscience Centre of the University of Göttingen, Structural Geology & Geodynamics, Goldschmidtstraße 3, 37077 Göttingen, Germany; 2 Ocean and Earth Science, University of Southampton, National Oceanography Centre, Southampton SO14 3ZH, UK

**Keywords:** Earth sciences, Geology, Applied geology, Methods in earth sciences, Petrophysics

## Abstract

Mohr diagrams are a simple and effective method that can help geoscientists consider, model, and predict the ranges of mechanical properties of rock, stresses, fluid pressures, and the resultant fractures that are likely to occur in the sub-surface. Mohr diagrams can be used to make predictions about how rocks may respond to change, with a transition from a stable state to fracturing occurring if there are changes in (1) the failure envelope, (2) stresses, and/or (3) fluid pressure.

This article uses Mohr diagrams to address two questions of significance to the energy transition. First, how will metasedimentary rocks, which are potential geothermal reservoir rocks, respond to thermal stimulation? Second, will fractures that may influence the underground storage of radioactive waste develop in a clay sequence during exhumation? Mohr diagrams are shown to be useful for highlighting misconceptions and input data problems, leading to improved understanding of how structures develop.

## Highlights

• Mohr diagrams are a simple and effective method
• They can be used to model rock properties, stresses, fluid pressures, and fractures
• Mohr diagrams highlight misconceptions and input data problems

## Graphical abstract

**Figure:**
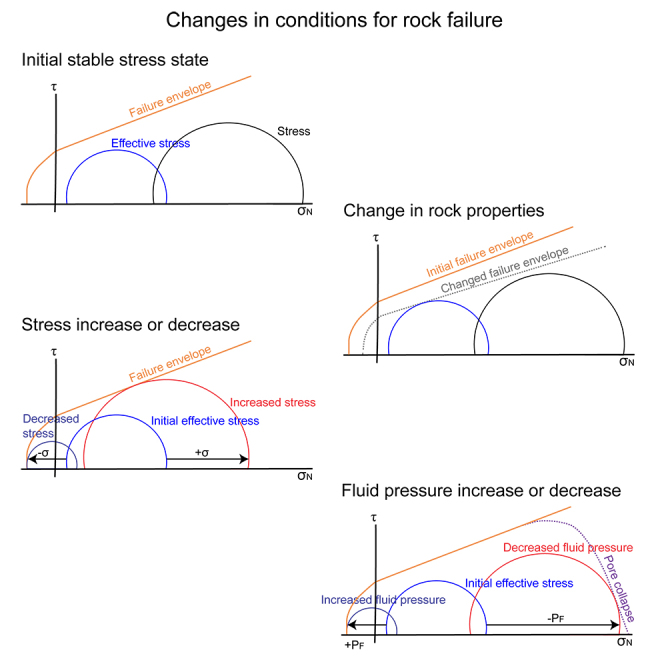

## Introduction

Knowledge about fractures and fluid flow is crucial to successful production of geothermal energy, carbon sequestration, and storage of radioactive waste. Sophisticated models of fractures and fluid flow in the subsurface, such as a discrete fracture network model,^1^^,^^2^^,^^3^ make various assumptions about rock mechanics and boundary conditions,^4^ and generally require input of data such as fracture apertures and connectivities^5^ and fluid flow rates.^6^ Such data are typically unavailable at an early stage of a project, when little or no well data are available. Furthermore, discrete fracture network modeling and similar techniques require the expertise, time, and software (and therefore expense) that may be unavailable to companies working on more sustainable energy production. Here, we discuss a simple method that helps in making predictions about fracturing, and that can help determine realistic input parameters for more sophisticated modeling techniques.

**Figure 1:**
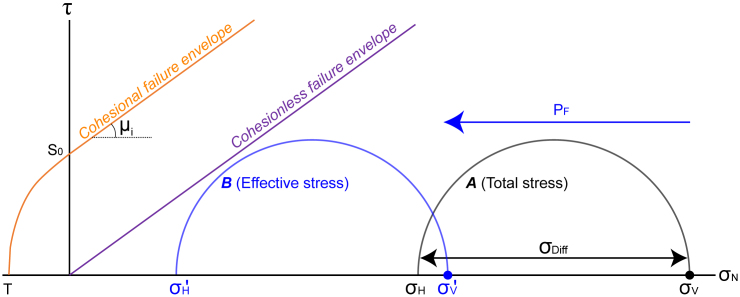
Mohr diagram for 2D stress showing the parameters used in this article Mohr diagrams are graphs of shear stress (τ) against normal stress (σ_N_). Here, the maximum compressive stress (σ_1_) is vertical (σ_V_), and the least compressive stress (σ_3_) is horizontal (σ_H_). Mohr circle *A* is the “total stress”, or the effective stress when fluid pressure (P_F_) = 0 and geostatic stress ratio (k_o_) = 0.5. Mohr circle *B* is the effective stress (σ′ = σ ˗ P_F_). Figure 2 shows the relationships between total stress, effective stress and k_o_. Two end-member failure envelopes are shown. The cohesional failure envelope is defined by the cohesion (S_0_), tensile strength (T) and the coefficient of internal friction (μ_i_). The cohesionless failure envelope is defined by μ_i_.

The Mohr diagram for stress (Figure 1) is a simple, commonly used graphical technique that has been used in structural geology and geomechanics for decades.^7^^,^^8^ There are several common uses of Mohr diagrams, which include:

*Conceptualizing* relationships between stresses, fluid pressure, rock properties, and fracturing.^9^ Mohr diagrams are therefore generally used in structural geology textbooks to illustrate these relationships.^10^^,^^11^

*Calculating* stresses, for example, on fault planes given knowledge of the orientations and magnitudes of the stress axes.^12^

*Constructing* failure envelopes from deformation tests.^13^

The aim of this article is to show how Mohr diagrams can be used to predict how rocks may respond to natural and man-made changes in sub-surface conditions. This is illustrated by dealing with two specific questions with broad application to geoenergy and the sustainable energy transition: (1) how reservoir rocks may respond to stimulation, using the example of modeling the thermal stimulation of a potential geothermal reservoir, and; (2) whether joints can develop in a clay during exhumation, discussed in terms of the underground storage of radioactive waste. In both questions discussed, we focus on how the effective stress might relate to some simple failure conditions, discussing the likelihood of brittle failure and the types (extensional or shear) of the resulting fractures. In most sub-surface situations, reliable estimates of depth, temperature and rock type can be made from wells and geophysical surveys, but there is generally much uncertainty in rock properties and stresses. To deal with these questions, and many other sub-surface issues, we need to develop a robust workflow. The paper also discusses how to handle variables, and how Mohr diagrams can be used to incorporate uncertainty, especially in the early stages of a project.

This article also discusses the interplay of external boundary stresses and internal fluid pressure in the creation of fractures. Mohr diagrams are used for clarity and simplicity, and we only discuss fracture mechanics and rock properties to the extent needed to account for the structural processes involved in rock fracture. Fracture mechanics considers fracturing to result from the development of stress concentrations around pores and small cracks in a material.^14^^,^^15^^,^^16^ We consider a spectrum of failure conditions ranging from intact rock, where fracture toughness and micro-flaws combine to determine the “strength” of the material, to fractured and granular materials, where frictional sliding dominates. We do not develop the ideas of fracture mechanics any further here, but refer readers to Engelder,^17^ Pollard and Fletcher,^15^ and Schultz^18^ for discussions of fracture mechanics in a geological context.

### Constructing Mohr diagrams

Mohr diagrams are a useful way of displaying relationships between stresses, fluid pressure, rock properties, and fracturing. This section shows the sequence in which we develop Mohr diagrams, and describes the input parameters used.

#### Mohr diagrams and stress axes

Mohr diagrams are graphs of shear stress against normal stress that allow determination of the stresses on planes of any orientation relative to the principal axes of stress (Figure 1; e.g.;^19^). They provide a convenient graphic representation of stress states and of failure.^10^ Shear stress (τ) is plotted on the y axis and normal stress (σ_N_) on the x axis. The principal axes of stress (σ_1_ = maximum compressive stress, σ_2_ = intermediate compressive stress, σ_3_ = minimum compressive stress) or principal axes of effective stress (σ′_1_ = maximum effective compressive stress, etc.) plot along the x axis. Note that, for simplicity, here we consider stress in 2D, with σ_2_ being ignored.^20^ It is often assumed^21^ that, in the upper few kilometers of the crust, one of the principal stresses is vertical (σ_V_). Here we consider σ_V_ and a horizontal stress (σ_H_). Any 2D state of stress can be represented by the Mohr circle, which intersects the x axis at σ′_1_ and σ′_3_. The center of the circle represents the mean effective stress (σ′_Mean_ = (σ′_1_ + σ′_3_)/2).

#### Components responsible for effective stress

We consider four components responsible for stress.

(1) The overburden stress (σ_V_) produced by the weight of the overlying column of rock, which is generally a function of depth and rock density. This (vertical) stress can be predicted with knowledge of rock densities, for example from wireline logs.^22^ σ_V_ = ρ_R_ z g, where: ρ_R_ = unit bulk weight of the rock, z = depth, g = gravity. Note, however, that non-lithostatic vertical stresses can occur if vertical stress is above or below that expected from the densities of the overlying rock.^23^^,^^24^ Such non-lithostatic vertical stresses have been attributed to bending of plates or other layers.^25^
(2) Fluid (or pore) pressure (P_F_) within pores and cracks in the rock, the rock usually being comprising mineral grains that may or may not be cemented together.
(3) Internally derived stresses, such as those arising from heating and cooling of rock, or such chemical changes as hydration or dehydration of clay minerals.^26^
(4) Externally applied loads, often categorized as tectonic stresses, that arise from variations in mass distribution and kinematic movement within the crust.

We therefore divide stresses into those that are externally applied (including by the overburden and by tectonic activity) and internally derived stresses (including chemical and thermal effects).

#### Fluid pressure and effective stresses

Deformation in brittle rocks is controlled by effective stress (σ′; e.g.,^27^^,^^28^). The vertical effective stress (σ_V_′) is σ_V_ minus fluid pressure (i.e., σ_V_′ = σ_V_ – P_F_), and in the situation in which the fluids are hydrostatically pressured is given by:

(Equation 1) $$\sigma_{V}^{\prime }=\left({\text{ρ}}_{R}-{\text{ρ}}_{W}\right)g\;z$$

where ρ_R_ = rock density, ρ_W_ = fluid density, g = gravitational acceleration, and z = depth. Terzaghi^29^ introduced the concept of effective stress to soil mechanics, and it has been widely applied to rock deformation since Hubbert and Rubey,^30^ who argued that thrust sheets slide on layers of over-pressured fluids that reduce the shear stress and therefore overcome friction. *Overpressure* is fluid pressure in excess of the *hydrostatic fluid pressure*, which is the fluid pressure to be expected in an equivalent column of water.^31^^,^^32^ Fluid pressure in the sub-surface can be measured using well log data^33^ and palaeo-fluid pressures can be estimated from such structures as veins.^34^

#### The geostatic stress ratio

Many textbooks and papers show increasing fluid pressure causing the Mohr circle to move to the left without changing diameter (i.e., the change in σ′_H_ = change in σ′_V_). Such representations are, however, unrealistic because the vertical stress and fluid pressure interact to cause a component of horizontal effective stress (σ′_H_∗), which, for uniaxial strain, is given by:

(Equation 2) $$\sigma_{H}^{\prime }*=k_{o}\;\sigma_{V}^{\prime }$$

where k_o_ is usually referred to as the *geostatic stress ratio*^35^ or *coefficient of lateral earth pressure*.^36^ σ′_H_∗ is therefore the component horizontal stress caused by the relationship between the vertical effective stress and the geostatic stress ratio, and it is used for the base cases in the modeling presented here. The geostatic stress ratio (k_o_) is the ratio of the base case horizontal effective stress (σ′_H_∗) to the vertical effective stress (σ′_V_). For an isotropic elastic material:

(Equation 3) $$k_{o}=\nu /\left(1–\nu \right)$$

where ν is Poisson’s ratio, which is generally in the range 0–0.5. Where ν = 0.5, the rock is incompressible and only then will the change in effective stress be the same in the vertical and horizontal directions. For most water-saturated rocks and soils, ν is in the range 0.15 < ν < 0.4.^37^ Poisson’s ratio is often thought of as a material parameter, but it is difficult to estimate for a rock because it increases with increasing porosity,^38^ fracture intensity (Z. Guo et al., 2012, SEG Technical Program Expanded Abstracts 2012, abstract), and with decreasing confining pressure.^39^ It also depends on the proportion of clay, water and organic material in the rock.^40^

**Figure 2:**
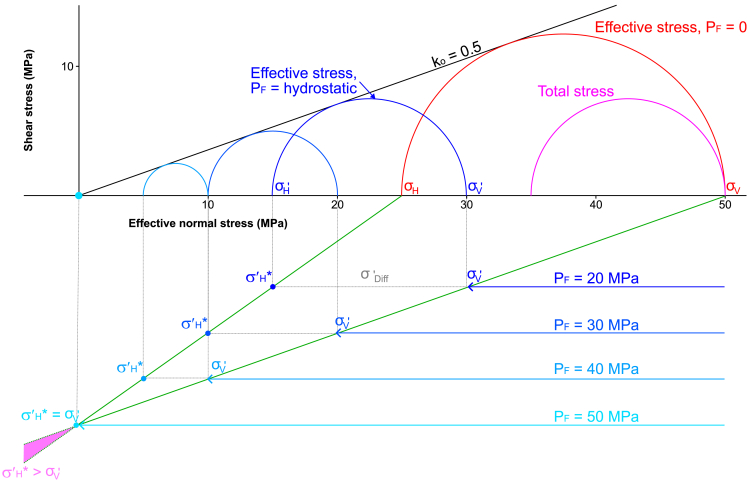
Mohr diagram illustrating the relationships between total stress, effective stress and geostatic stress ratio (k_o_) The Mohr diagram is based on the following: (1) σ_V_ = σ_1_; (2) k_o_ = 0.5; (3) uniaxial strain; (4) an average rock density of 2.5 g/cm^3^; (5) depth of 2 km; (6) no applied horizontal (e.g., tectonic or thermal) stresses. This means that σ_V_ = 50 MPa and σ_H_ = 25 MPa (red Mohr circle). Hydrostatic fluid pressure (P_F_ = 20 MPa) produces σ_V_′ = 30 MPa and σ_H_′ = 15 MPa, so the differential effective stress (σ′_Diff_ = σ_1_′ ‒ σ_3_′) is therefore smaller that the differential stress (σ_Diff_ = σ_1_ ‒ σ_3_). The lower part of the figure shows how differential effective stress decreases as fluid pressure increases (blue Mohr circles) until the effective stresses are zero when P_F_ = σ_V_. The condition of uniaxial strain predicts that when P_F_ > σ_V_, σ_V_′ < σ_H_′ (pink area).

Figure 2 shows how k_o_ is used in the modeling presented in this article, showing the change in effective stresses as fluid pressure increases. In this case, σ_V_ = σ_1_, k_o_ = 0.5 and the rocks are laterally confined (i.e., uniaxial strain). Assuming a mean overburden density of 2.5 g/cm^3^ and a depth of 2 km, the overburden stress (σ_V_) = 50 MPa. Under hydrostatic fluid pressure, σ′_V_ = 30 MPa (where P_F_/σ_V_ = λ = 0.4, based on the assumption that P_F_ = 20 MPa). Using k_O_ = 0.5, would give σ′_H_∗ = 15 MPa under hydrostatic conditions. The differential effective stress (σ′_DIFF_) decreases as the fluid pressure increases, with σ′_V_ = σ′_H_ = zero when P_F_ = σ′_V_. Note that the model predicts that, under these boundary conditions, σ′_H_∗ exceeds σ′_V_ when P_F_ > σ_V_ (pink area in Figure 2). The model therefore predicts the development of horizontal extension fractures when P_F_ > σ_V_, even if σ_1_ = σ_V_. The effects of fluid pressure and k_o_, including the development of horizontal extension fractures when P_F_ > σ_V_, is discussed in more detail by Bons et al.^9^ Segura et al.^41^ show the effects of k_o_ on stresses within reservoirs caused by depletion of fluid pressure.

We start out modeling with a base case of uniaxial strain, in which there is no horizontal strain and the only horizontal stress is given by σ′_H_∗ (Equation 2). We use this as a starting point to consider other sorts of stress changes, such as those induced by thermal or tectonic effects.

#### Tectonic and thermal stresses

While the base case models assume σ′_H_ = σ′_H_∗, we can increase the horizontal compressive stress to model the effects of tectonic compression or an increase in temperature, or decrease the horizontal compressive stress to model the effects of tectonic tension or a decrease in temperature. The stress change (Δσ_T_) for a change in temperature (ΔT) is given by:

(Equation 4) $$\Delta \sigma_{T}^{\prime }=\left(\alpha \;E‒\Delta T\right)/\left(1‒\nu \right)$$

where α = coefficient of thermal expansion, E = is Young’s modulus, and ν = Poisson’s ratio.^10^^,^^42^^,^^43^ α varies from ∼3 K^−1^ to 30 × 10^−6^ K^−1^ (typically around 10^−5^ K^−1^) for most rocks. Using E = 60 GPa and ν = 0.25 gives Δσ/ΔT = 0.8 MPa K^−1^. Lowering the temperature by only 10 K could therefore induce a horizontal stress change of ∼8 MPa, so these thermal stresses can be significant.^44^

#### Rock properties and failure envelopes

Mohr diagrams are useful for illustrating the conditions under which fracturing may occur in a particular rock under specific conditions (i.e., the *failure envelope*; Figure 1). Failure envelopes can be constructed for a particular rock type using the results of triaxial rock deformation tests.^45^ The failure envelope can be constrained in a simple way using the cohesion (S_0_), tensile strength (T) and coefficient of internal friction (μ_i_), as shown by Sibson and Scott.^46^ Here, we use values of S_0_, T and μ_i_ from the literature that we consider are appropriate for the rock types being modeled. Two end-member failure envelopes are used (Figure 1). The *cohesional failure envelope* is used for rocks that have a cohesional and tensile strength. The *cohesionless failure envelope* is used for granular materials (soils and unconsolidated sediments with no cohesion or tensile strength, or for rocks that contain cohesionless fractures.^47^

#### Fracturing and fracture types

Fractures in rock have been studied for over 400 years^48^ and are almost ubiquitous in the upper crust, particularly in rocks exposed at the surface. They control a wide range of physical properties, including rock strength and stiffness,^49^ the transport of fluids in rock^50^ and heat transfer in the crust.^51^ Although a full understanding of fracture in rock materials requires consideration of stress concentrations around flaws (modern fracture mechanics, e.g.,^18^), we adopt a simplified approach here. Fracturing is classically attributed to conditions where the stress components exceed some critical value, usually termed the *strength*, which is thought to be a property of the material.^52^ Tensile failure occurs if:

(Equation 5) $$\sigma_{3}^{\prime }\leq T$$

where T is the tensile strength. We use positive for compressive stresses, so the effective minimum stress must be lower (and negative) than the tensile strength of the rock (which will also be negative). Shear failure occurs if:

(Equation 6) $$\tau \geq S_{0}+{\text{μ}}_{i}\;\sigma_{N}$$

where μ_i_ is the coefficient of (internal) friction and S_0_ is the cohesion, and these conditions are usually linked to produce a *failure envelope*^53^^,^^54^ (Figure 1). For most rocks, T ≪ – 100 MPa, with soils and poorly-consolidated sediments having T → 0, whereas for shear failure 0.5 < μ_i_ < 1 (generally) and 0 < S_0_ < 100 MPa.^55^ Values of T ≈ – 2 MPa, S_0_ ≈ 4 MPa and μ_i_ ≈ 0.75 are typical of intact sandstone or shale,^56^^,^^57^ while crystalline rocks tend to have tensile strengths of > – 10 MPa.

For the purposes of this article, we assume a lower bound to likely failure envelopes is given by the failure envelope shown by the “Cohesionless” failure envelope (Figure 1), which is typical of slip on pre-existing fractures and failure of soils. Such failure envelopes typically have coefficients of sliding friction of 0.6–0.9 (average 0.75; e.g.,^56^).

**Figure 3:**
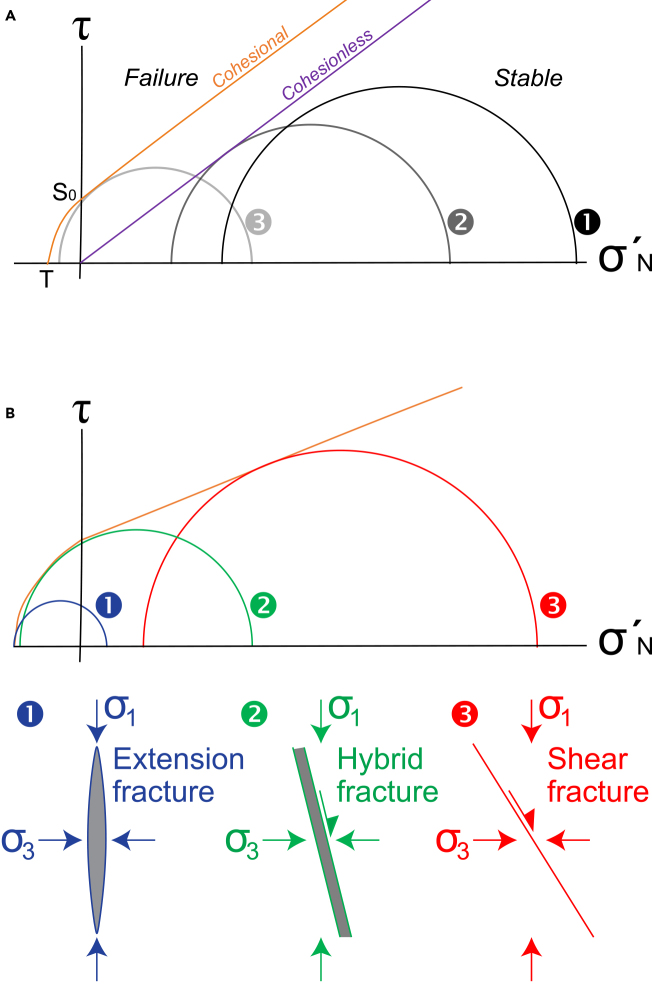
Schematic Mohr diagrams of effective stresses and fracture types (A) Mohr diagram of shear stress (τ) against normal effective stress (σ_N_′) (Figure 1B in Peacock et al.^58^). Continuous line (“cohesional”) = failure envelope for an intact rock, with tensile strength (T) and cohesion (S_0_). Three stress states are shown, representing stable (➊), shear failure on fracture or cohesionless soil (➋) and intact rock (➌). (B) Schematic Mohr diagram showing the fields in which extension (➊), hybrid (➋) and shear (➌) mode fractures occur.^59^

Figure 3A shows different stress states and how they relate to fracturing. Stress state ➊ is in the stable region, so no fracturing would occur. Stress state ➋ would cause slip on a suitably orientated pre-existing fractures or failure in cohesionless soil. Stress state ➌ would cause fracturing of intact rock.

The type and orientation of fractures developed depends on the relationship between the Mohr circle for effective stress and the failure envelope (Figure 3B). Extension fractures typically develop perpendicular to the direction of σ′_3_ if σ′_3_ touches the failure envelope on the τ = 0 axis (Figure 3B➊), which generally requires a relatively low differential stress (σ_Diff =_ σ_1_ – σ_3_). Shear fractures generally develop if the Mohr circle touches the failure envelope in the compressive field (Figure 3B➌), this typically requiring a relatively high σ_Diff_. Hybrid fractures develop by synchronous extension and shear modes,^59^ and occur if the Mohr circle touches the curved part of the failure envelope within the tensile field of the Mohr diagram (Figure 3B➋).

#### Changing conditions for fracturing

**Figure 4:**
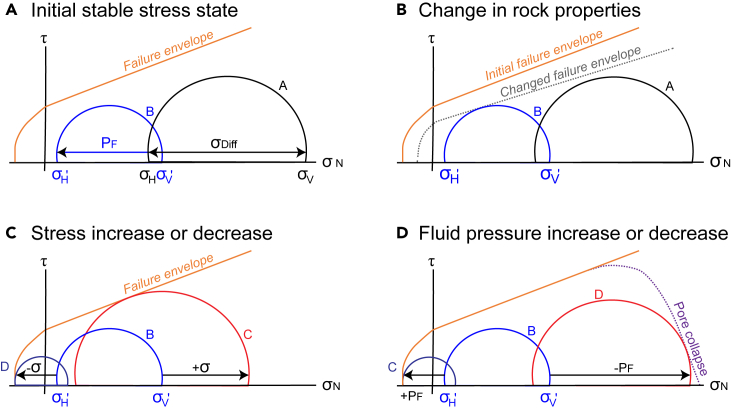
Mohr diagrams showing the relationships between rock properties, stresses (in 2D), fluid pressure (P_F_) and failure τ = shear stress, σ_N_ = normal stress, σ' = effective stress, σ_Diff_ = differential stress. Again, σ_1_ = σ_V_ and = σ_3_ = σ_H_. (A) An applied stress (A) and fluid pressure (P_F_) combine to produce an effective stress (B) that is in the stable regime. (B) Fracturing can occur if the rock properties change, such that a different failure envelope applies that enables failure. (C) Fracturing can occur if there is an increase (C) or decrease (D) in the stresses.^60^ (D) Fracturing can occur if there is an increase (C) in the fluid pressure, while pore collapse may occur if there is a decrease (D) in the fluid pressure.^61^

In this article, we use Mohr diagrams to illustrate the conditions under which a rock can go from a stable stress state (i.e., no active fracturing occurs; Figure 4A) to an unstable stress state (i.e., fracturing occurs). In the stable state, the magnitudes of the maximum (σ′_1_) and minimum (σ′_3_) effective stresses are such that the Mohr circle does not touch the failure envelope for a particular rock (➊ in Figure 3A). This condition changes with.

• Changes in *rock properties*, such as changes in the tensile strength (T), cohesion (S_0_) and coefficient of internal friction (μ_i_) (Figure 4B; e.g., Scholz^62^). Rock properties can change, for example during diagenesis and cementation.^63^
• Changes in the *stress state* (σ), usually defined in terms of the principal stresses, mean stress and differential stress.^10^ Stresses are in turn controlled by factors such as depth of burial (overburden), tectonic (horizontal) stresses and other change in the physical state of the material, such as expansion or contraction caused by temperature and volume change.^64^ Changes in stresses that lead to fracturing (Figure 4C) can either be by increasing the applied compressive stresses,^65^ or by reducing the applied compressive stresses.^66^
• Changes in the *fluid pressure* (P_F_) in the pores and cracks ^67^ (Figure 4D). Changes in fluid pressure that can lead to fracturing can either be an increase in fluid pressure^68^ or a reduction of fluid pressure, which can cause pore collapse.^61^^,^^69^

#### Rationale

Mohr diagrams have been used for the following reasons. First, Mohr diagrams and simple equations for rock behavior enable analysis to be performed using a spreadsheet. It is a rapid and inexpensive technique that can be easily adapted to a range of situations in which predictions may need to be made about fracturing in the sub-surface. For example, it is straightforward to change the input parameters as more realistic values become available as exploration proceeds. Second, the approach is good for testing the potential effects of different parameters, such as how rocks with different mechanical properties may respond differently to thermal stimulation. Third, the task of deciding which input values to use helps us recognize what we do and do not know about the potential reservoir rocks. For example, a range of Young’s moduli are used in this article in the absence of specific data from the reservoir rocks.

### Simplifying assumptions and model set-up

Several assumptions are made to simplify the modeling.

• It is assumed that the intermediate stress axis does not influence deformation, so that two-dimensional Mohr diagrams for stress are used, with a vertical (σ_V_) and a horizontal stress (σ_H_). While this assumption may be unrealistic in some circumstances,^12^ it does simplify the analysis and is fit-for-purpose for the objectives stated in this manuscript.
• An Andersonian stress state^21^ is assumed, with one stress vertical (σ_V_). Here, we use σ_1_ = σ_V_. While non-Andersonian stress systems can occur,^70^ Andersonian stresses are typical of conditions in the upper crust and this assumption is commonly used.^71^
• The vertical stress is given by the mass of the overlying rocks (overburden stress), with appropriate average densities used here (e.g., Table 1).

**Table 1:** Parameters used for the modeling of thermal stimulation of Devonian quartzites and slates at Havelange

Parameter	Quartzite, minimum ΔT	Quartzite, maximum ΔT	Slate	Unit
**Depth (z)**	5,000	5,000	5,000	m
**Average rock density (ρ**_**R**_**)**	2.68^a^	2.68^a^	2.68^a^	g/cm^3^
**Overpressure**	0	0	0	MPa
**Poisson’s ratio (ν)**	0.09^b^	0.21^b^	0.22^c^	
**Applied tectonic stress (σ**_**T**_**)**	0	0	0	MPa
**Cohesion (S**_**0**_**)**	39.5^d^	83^d^	30^d^	MPa
**Coefficient of internal friction (μ**_**i**_**)**	0.82^d^	1.27^d^	0.84^c^ and 0.63^d^	
**Tensile strength (T)**	20	41.5	15	MPa
**Coefficient of thermal expansion (α)**	20^e^	10^e^	8^f^	10^−6^ K^−1^
**Young’s modulus (E)**	91^b^	35^b^	12 and 56^a^	GPa
*Vertical stress (σ*_*V*_*)*	131.5	131.5	132.40	MPa
*Fluid pressure (P*_*F*_*)*	49.1	49.1	49.1	MPa
*Geostatic stress ratio (k*_*o*_*)*	0.1	0.27	0.283	
*Vertical effective stress (σʹ*_*V*_*)*	82.4	82.4	83.4	MPa
*Horizontal effective stress (σ′*_*H*_*∗ = σ*_*V*_*k*_*o*_*)*	8.2	22.2	23.6	MPa

Input parameters are in bold and output parameters are in italics. Two end-member cases are considered for the quartzites, these having the values that would require the minimum temperature decrease (“Quartzite, minimum ΔT” column) and the values that would require the largest change in temperature (“Quartzite, maximum ΔT” column). Two end-member values of Young’s modulus are used for slates, but other values not changed. Data sources.

a McNamara et al.^72^

b Davarpanah et al.^73^

c Cai et al.,^74^ who give friction angles of 30–50° for slate, and we use 40° (i.e., μ_i_ ∼ 0.84).

d Bär et al.^75^ (table 19).

e Wang et al.^76^ quote a value of 11.75 10^−6^ K^−1^.

f Lee et al.^77^ For tensile strength, we use 50% of the cohesion. Note that values will tend to vary with angle between stresses and cleavage. For example, Gholami and Rasouli^78^ give cohesion values for slate of 64 MPa when σ_1_ is perpendicular of cleavage and 11 MPa when σ_1_ is at 30° to cleavage.
• It is assumed that rocks are brittle, with deformation governed by the failure envelope appropriate for that rock type.
• Similarly, it is assumed that the rock properties, derived from rock deformation tests and reported in the literature, are representative of the conditions studied. This assumption may be unrealistic because short-term laboratory tests on relatively small rock samples tend to ignore the effects of pre-existing fractures and, thus, overestimate the strength of a rock mass.^79^
• We consider a reference (i.e., initial base-case) state where the horizontal strains are zero (the *uniaxial strain* condition; e.g.,^80^). The rocks are therefore assumed to be laterally constrained, meaning that the Poisson’s ratio and geostatic stress ratio give the effect of the overburden on the horizontal stress. While this is a commonly made assumption,^81^^,^^82^ it will not apply in rocks undergoing horizontal extension or contraction.^83^

We start each model with a simple base-case that has the following characteristics. Firstly, the initial horizontal stress is related simply to the overburden stress and the Poisson’s ratio. There is no applied horizontal (e.g., tectonic or thermal) stress. This means that, in the absence of applied tectonic or thermal horizontal stresses, the horizontal stress is given by Equation 2. Note that the application of thermal horizontal stresses is modeled in the next section. Also note that this assumption is made in the absence of information about the orientations and magnitudes of the horizontal stresses. The model can be updated and improved if and when measurement of *in situ* stresses become available. Secondly, the fluid pressure is hydrostatic. Both of the situations modeled involve changes in the magnitudes of the horizontal stress, temperature and/or fluid pressure to predict conditions under which failure will occur.

## Results

### Modeling thermal stimulation

#### Question and background

The question being addressed in this section is: how are metasedimentary rocks (specifically quartzites and slates) in a potential geothermal reservoir likely to respond to thermal stimulation? The aim is to predict the decreases in rock temperature that would be needed to generate the tensile thermal stresses necessary to reactivate existing fractures in extension or shear modes, or to generate new fractures (thermal cracking).

**Figure 5:**
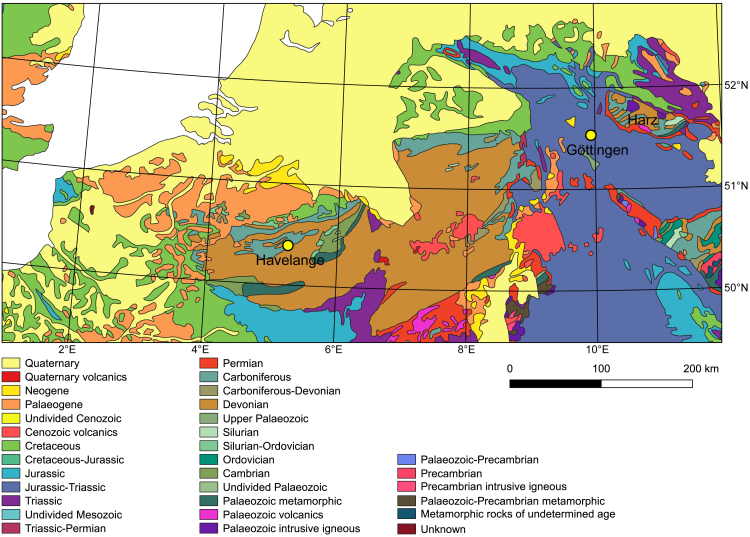
Geological map of parts of western Europe, showing the locations of Havelange (50°18′0.99″N, 5°14′49.51″E), the Harz Mountains (51°47′N, 10°36′E) and Göttingen (51°32′N, 9°56′E) These areas were used in the MEET project^84^ to explore the geothermal potential of Upper Paleozoic metasedimentary rocks.

A gas exploration borehole was drilled at Havelange, Belgium (50°18′0.99″N, 5°14′49.51″E; Figure 5), in the early 1980s.^85^ No economic oil or gas reserves were discovered, but the borehole is now being used as part of a study to test the viability of the Devonian metasedimentary rocks as hosts for geothermal reservoirs. The work presented here was done as part of the European funded project MEET (Multidisciplinary and multi-context demonstration of EGS exploration and Exploitation Techniques and potentials) to develop enhanced geothermal systems (EGS) across Europe.^86^^,^^87^^,^^88^^,^^89^ One aim of the MEET project was to develop a stimulation strategy for Havelange based on limited data from the borehole.

The MEET project considered three stimulation methods for at Havelange: thermal,^90^ chemical^91^ and hydraulic.^92^ Here, we describe the results of modeling the potential effects of thermal stimulation. Note that Peacock et al.^58^ use Mohr diagrams to predict the effects of hydraulic stimulation for the Devonian and Carboniferous metasedimentary rocks that are expected to occur beneath Göttingen (Figure 5).

Cooling gives rise to thermal contraction and heating gives rise to thermal expansion. The resulting stress change (ΔσT) for a change in temperature (ΔT) is given by Equation 4. Cooling joints are an example of structures caused by thermal cooling. They are well known from intrusions of hot magma into cooler rocks^93^^,^^94^ and from extrusions.^95^ It has also been suggested^17^^,^^96^ that regional cooling during exhumation can be the cause of many natural joint systems.

#### Available data

Limited data specific to the well at Havelange are available. Leiss et al.^97^ give information about the lithologies and the fractures they contain from the borehole at Havelange and from exposed analogues. Cabidoche et al.^98^ use mud-loss and dipmeter data to suggest that open fractures occur in the Pragian (Lower Devonian) quartzites, which are the main targets for geothermal exploration at Havelange. Limited data about temperatures in the borehole at Havelange are available, being reported to be “119°C (not corrected)” at 5370 m measured depth.^97^ In the absence of mechanical data for Havelange, literature data are used for the general mechanical properties of the rocks seen in the borehole and in exposed analogues. Parameters used are shown in Table 1. Published results for stimulation of Precambrian quartzites at the Raft River geothermal site (Utah, USA) are also available for comparison.^99^^,^^100^

Note that limited information is available about the orientations or magnitudes of the horizontal stresses. While the World Stress Map^101^ indicates the maximum horizontal stress in the region is orientated approximately NW-SE, normal, strike-slip and thrust regimes are all reported in the region. Limited information is available about the rock mechanical properties, formation pressures or about fluid chemistry in the sub-surface. The modeling presented here therefore uses published values for the mechanical properties of similar rocks (Table 1), along with various starting assumptions (e.g., no applied tectonic stresses, fluid density of 1 g/cm^3^).

#### Model set-up

The Devonian sequence at Havelange consists of a variety of rock types, including quartzites, sandstones and slates.^98^ Here we compare the quartzites and the slates, with the parameters used being shown in Table 1, to discuss thermal stimulation. For example, while we consider the values selected to be reasonable end-member values for a quartzite, we acknowledge that actual values outside these ranges occur, with Reeher et al.^102^ showing cohesion (S_0_) = 103 MPa. The results therefore need to be treated with caution.

Two starting-point (“base case”) models are used for Devonian quartzites, with the parameters used shown in Table 1. The ranges of values were chosen to show reasonable spreads of values, with one end-member base case requiring the smallest change in temperature to cause thermal stimulation (“Quartzite, minimum ΔT” in Table 1) and the other end-member base case requiring the largest change in temperature to cause thermal stimulation (“Quartzite, maximum ΔT” in Table 1). For Devonian slates, the starting points are base cases with two end-member values of Young’s modulus (Table 1). The steps taken are.

(1) Create the end-member “base case” Mohr diagrams using the data presented in Table 1.
(2) Determine the changes in horizontal stress needed to start reactivating (either in shear or dilation mode) cohesionless fractures with favorable dips, or to generate new fractures in intact rock.
(3) Calculate the changes in temperature needed to cause those changes in horizontal stress (i.e., the thermal stresses, σ_THERM_). Note that the vertical stress will not change during changes in temperature because the overburden does not change.

The relationship between the changes in temperature (ΔT) and thermal stress (σ_THERM_) is given by Equation 4. Note that quartz has the highest α of any common mineral, so rocks with high quartz content have high α.^103^ By rearranging Equation 4, we obtain the temperature change needed for inducing a certain thermal stress:

(Equation 7) $$\Delta T=\left(\sigma_{\text{THERM}}/\left(\alpha \;E\right)\right)\times \left(1–\nu \right)$$

Depth and rock density control the vertical (overburden) stress and, therefore, influence the magnitude of the horizontal stress.

#### Assumptions

The assumptions used in this article are given in an earlier section, but the following additional assumptions are made for the modeling of thermal stimulation at Havelange.

(1) The two-dimensional stress state, with the vertical stress and a horizontal stress is sufficient for modeling. The magnitudes and orientations of the stresses at Havelange are unknown, so using a three-dimensional stress state would require additional assumptions.
(2) The mechanical properties given in Table 1 are applicable to the rocks at a depth of 5 km in the Havelange borehole. Ranges of values that are considered representative of quartzite are used, with two different end-member values of Young’s modulus being considered for the slates.
(3) For simplicity, slates are considered as being homogeneous and isotropic. It is acknowledged, however, that slates are anisotropic. For example, the orientation of cleavage relative to the applied stresses has a strong effect on slate strength.^104^
(4) Fluid pressure is hydrostatic with a static water table near surface. This seems reasonable because neither overpressure nor underpressure have not been reported from the borehole.
(5) The rocks around the borehole are cooled in a homogeneous way in a homogeneous stress field. We do not consider heterogeneous cooling, or the circumferential compressive stresses and hoop stresses around the borehole wall.

#### Results of the modeling

The modeling suggests the following for the quartzites at a depth of 5 km.

**Figure 6:**
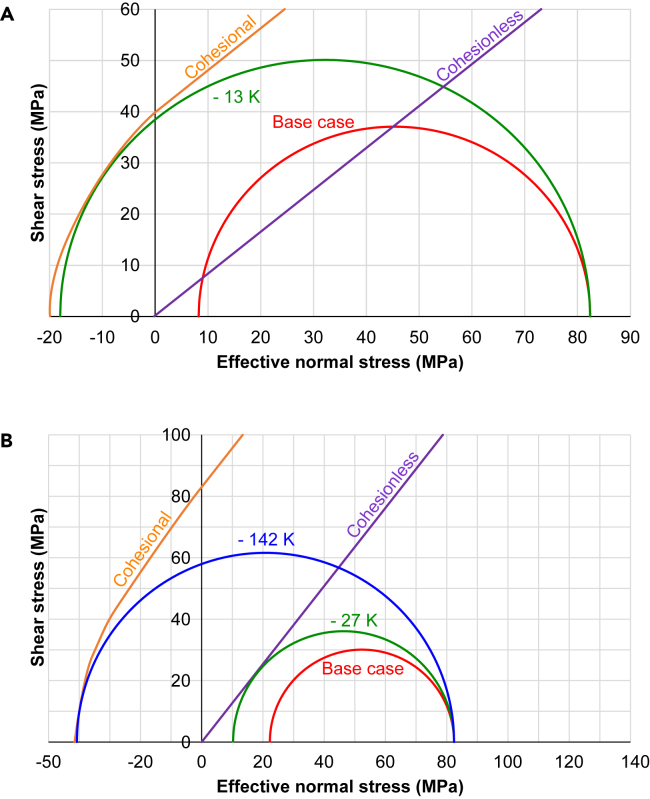
Mohr diagrams for quartzites at Havelange showing the effects of decreases in thermal stress related to thermal contraction (A) The end-member case requiring the minimum temperature decrease to cause fracturing (“Quartzite, minimum ΔT” column in Table 1). The model predicts that favorably dipping cohesionless fractures (e.g., joints with dips of ∼70°) would be reactivated in shear mode without a decrease in temperature. A temperature decrease of only 13 K (green Mohr circle) would be enough to generate new hybrid fractures. (B) The end-member case requiring the maximum change in temperature to cause fracturing (“Quartzite, maximum ΔT” column in Table 1). A temperature decrease of about 27 K (green Mohr circle) will start to reactivate favorably dipping cohesionless fractures in shear mode. A temperature decrease of about 142 K (blue Mohr circle) is predicted to generate extension or hybrid fractures if there are not pre-existing cohesionless fractures.
(2) In the case requiring the smallest decrease in temperature (Figure 6A), a decrease in horizontal stress of ∼26 MPa would be enough to generate new hybrid fractures in a cohesional quartzite. This decrease in horizontal stress of ∼6 MPa would require a temperature decrease of ∼26 K. Note, however, that Cabidoche et al.^98^ suggest that open fractures exist within the potential reservoir, so stimulation is more likely to reactivate existing cohesionless fractures than to generate new fractures.
(3) In the case requiring the largest decrease in temperature (Figure 6B), a decrease in horizontal stress of ∼12 MPa would cause favorably dipping cohesionless fractures to be critically stressed and subject to shearing. This would require a temperature decrease of ∼27 K. Cohesionless fractures with dips of ∼70° are likely to be reactivated first, and reactivation will be in shear mode.
(4) If there are no pre-existing cohesionless fractures, a decrease in horizontal stress of ∼63 MPa would be needed to generate new extension or hybrid fractures (i.e., combined extension and shear modes^59^), this requiring a temperature decrease of ∼142 K. These extensional or hybrid fractures are predicted to have steep dips.

(1) In the base case requiring the smallest decrease in temperature (Figure 6A), favorably dipping cohesionless fractures would be critically stressed and subject to shearing in the present stress state and without cooling. This suggests that the lowest end-member value selected for k_o_ is too low (i.e., that the lower value of Poisson’s ratio used is probably too low) or that the assumption of no applied tectonic compressional stress is incorrect. Cohesionless fractures with dips of ∼70° are likely to be reactivated first. In the base case requiring the largest decrease in temperature (Figure 6B), however, the rocks would currently be in a stable stress state, without reactivation of existing fractures or the generation of new fractures.

These results therefore suggest both that reactivation of pre-existing fractures may occur with relatively small changes in temperature, and that the selected lower end-member value for k_o_ (i.e., Poisson’s ratio) is too low or that there is an applied tectonic compressional stress.

The feasibility of thermal stimulation of quartzites in EGS reservoirs is illustrated by Bradford et al.,^99^^,^^100^ who present results of thermal stimulation of Precambrian quartzites at a depth of ∼1,643 m in an EGS site in Idaho, USA. They show that water injected at temperatures of between 29°C and 39°C caused an increase in flow rates in a reservoir with a mean temperature of 195°C (i.e., thermal stimulation was observed).

**Figure 7:**
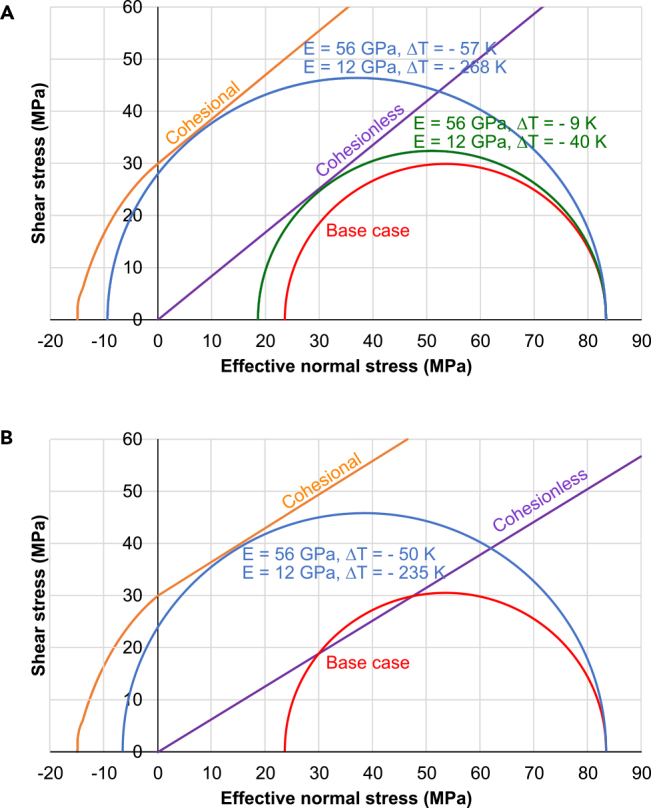
Mohr diagram for slates at Havelange showing the effects of decreasing temperature for both both a Young’s Modulus of 12 GPa and of 56 GPa (A) Based on the base case parameters shown in Table 1 (red Mohr circle), including a coefficient of internal friction of 0.84,^74^ shear on existing cohesionless fractures (green Mohr circle) would require a tensile thermal stress of about 5 MPa, which would require a temperature decrease of about 40 K if the Young’s modulus was 12 GPa, and a temperature decrease of about 9 K if the Young’s modulus was 56 GPa. The generation of new shear fractures would require a temperature decrease of about 268 K if the Young’s modulus is 12 GPa, and a temperature decrease of about 57 K if the Young’s modulus is 56 GPa. (B) Using a coefficient of internal friction of 0.63,^75^ favorably orientated cohesionless fractures would be unstable in the base case model, but the generation of new shear fractures would require a temperature decrease of about 235 K if the Young’s modulus is 12 GPa, and a temperature decrease of about 50 K if the Young’s modulus is 56 GPa.

Figure 7 shows model results for the slates at Havelange. Note that two different values of the coefficient of internal friction from the literature are used to illustrate problems of finding appropriate values for the parameters. Figure 7A uses a coefficient of internal friction that seems reasonable from the results of Cai et al.,^74^ while Figure 7B uses the one value of coefficient of internal friction for the slates at Havelange presented by Bär et al..^75^ The results do not appear to be strongly influenced by these different values for the coefficient of internal friction. The results presented in Figure 7 suggests that slates would require lower temperatures for thermal stimulation (i.e., more cooling) than would quartzites. This is because slates tend to have lower Young’s moduli and coefficients of thermal expansion than quartzites. It also suggests that thermal stimulation of slates would tend to reactivate existing cohesionless fractures in shear mode, or produce new shear fractures, rather than the new hybrid fractures predicted for quartzites. Note, however, that there is an overlap between the predicted changes in temperature required for thermal stimulation for the quartzites and slates. The values for temperature change for quartzite shown in Figure 6B are greater than the values for slates with E = 56 GPa shown in Figure 7. This suggests that it is possible that quartzites and slates may respond in a similar way to thermal stimulation.

#### Validity and how the modeling can be improved

The predicted values of temperature decrease need to be treated with caution. For example, smaller temperature decreases would be needed if there is an applied tensile tectonic stress, and because injection of cooler fluids would probably require an increase in fluid pressure (although ideally below pressures that would cause fracturing of overlying rocks). Also, it is acknowledged that the temperature changes predicted in the model are unlikely to equate in a simple way to the temperatures of the fluids needed to cause the changes predicted. For example, those fluids are unlikely to change the temperature of the rock volume uniformly. The results do, however, indicate what may happen during thermal stimulation.

The validity and accuracy of the modeling approach depend both on the assumptions used and the quality of the input data. These can be improved, either by making improved predictions of the *in-situ* stresses,^105^ or as more and better borehole data become available. This includes accurate data on the mechanical properties of the reservoir rocks, on the structures they contain, and on the *in-situ* stresses.

### Modeling joint development in clay

#### Question and background

The question being addressed in this section is: how much exhumation would be needed to generate joints in the Opalinus Clay? The work presented here has been done as part of a project to identify potential sites for the underground storage of radioactive waste in Germany, with the Opalinus Clay being a potential target horizon.^106^ The Opalinus Clay is modeled to predict whether seal integrity may have been compromised by uplift-related joints. The aim is to predict the exhumation-related decreases in vertical stress and temperature, and the related decrease in horizontal stresses, that may generate extension fractures in the Opalinus Clay. Joint development has been related to exhumation by various authors,^107^^,^^108^ with some authors suggesting joints are caused by poroelastic relaxation during exhumation.^109^^,^^110^ Akker et al.^111^ describe joints in the Opalinus Clay at the Mont Terri Rock Laboratory in Switzerland, but state that they are rare. Akker et al.^111^ also describe faults with displacements typically not exceeding the drill core diameter.

#### Available data

The parameters used for the modeling are shown in Table 2, where specific references are given. These data are derived from publications about the Mont Terri Rock Laboratory. Note the following about the data in Table 2.

• Lisjak et al.^116^ show that the uniaxial compressive strength of Opalinus Clay is controlled by the angle between loading and anisotropy, with a maximum of 14 MPa when loaded perpendicular to anisotropy and a minimum of 3.5 MPa when loading is at 45° to anisotropy. We use cohesion (S_0_) = 14 MPa to model the situation in which maximum compressive stress is vertical and anisotropy is horizontal.
• Schuster et al.^112^ state that samples deformed at 45° and 90° to anisotropy show a coefficient of internal friction of ≈0.31, and samples deformed parallel to anisotropy show a coefficient of internal friction of ≈0.44. We use 0.31, to model maximum stress perpendicular to anisotropy. Schuster et al.^112^ also state that they *assume* (without explanation) a Poisson ratio of 0.25.

**Table 2:** Parameters used for the modeling of joint development in the Opalinus Clay, starting with a maximum burial depth of 2 km below the ground surface

Parameter	Opalinus Clay	Unit
**Maximum burial depth**	2,000	m
**Average density**	2.37^a^	g/cm^3^
**Fluid density**	1	g/cm^3^
**Overpressure**	0	MPa
**Poisson’s ratio (ν)**	0.25^a^	
**Applied tectonic stress**	0	MPa
**Cohesion (S**_**0**_**)**	3.6^b^	MPa
**Coefficient of internal friction (μ**_**i**_**)**	0.47^b^	
**Tensile strength (T)**	1.8^c^	MPa
**Coefficient of thermal expansion (α)**	19^d^	10^−6^ K^−1^
**Young’s modulus (E)**	10.49^a^	GPa
*Vertical stress at maximum depth*	46.5	MPa
*Fluid pressure at maximum depth*	19.62	MPa
*Geostatic stress ratio (k*_*o*_*)*	0.333	
*Vertical effective stress (σ′*_*V*_*) at maximum depth*	26.88	MPa
*Horizontal effective stress (σ′*_*H*_*= σ′*_*V*_*k*_*o*_*) at maximum depth*	8.95	MPa

Input parameters are in bold and output parameters are in italics. The geothermal gradient is assumed to be 30 K per km. The data are from the Mont Terri Rock Laboratory in Switzerland, where the structural history is different from the region of interest in Germany (see text). Data sources.

a Schuster et al.^112^ (mean values from 9 samples).

b Bossart,^113^ with the coefficient of internal friction based on an internal friction angle of 25°.

c Jobmann et al.^114^ (mean values from 6 samples).

d Jobmann and Polster.^115^ Maximum depth, overpressure and applied tectonic stress are starting point values used in the base case.

See Khaledi et al.^117^ for a recent account of triaxial tests of the Opalinus Clay from Mont Terri.

#### Model set-up

**Figure 8:**
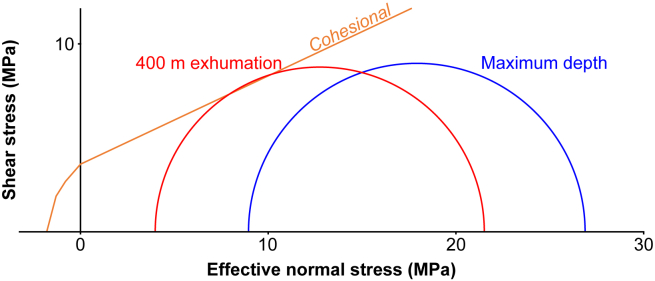
Mohr diagram for the Opalinus Clay with a maximum burial depth of 2 km and no overpressure The Mohr circles show the depth of 2 km (blue), and 400 m exhumation (red), developing shear fractures in cohesional material (red) (based on the data presented in Table 2). The differential stress decreases from 17.93 MPa at 2 km to 17.53 MPa after 400 m of exhumation because the effect of the geostatic pressure ratio is partly balanced by the effect of thermal cooling. The model predicts the development of shear fractures rather than joints, because of the low cohesion and the low coefficient of internal friction of the Opalinus Clay.

The model shown in Figure 8 is based on the data presented in Table 2. The maximum burial depth is modeled as being 2 km, there is no overpressure, and the geothermal gradient is 30 K per km. The reduction of overburden is associated with decreases in vertical stress, as well as the component of horizontal effective stress related to the interaction between vertical stress and fluid pressure (σ′_H_∗; Equation 2). Cooling also causes a horizontal tensile stress (Equation 4). The amount to exhumation required to cause the changes in stress necessary for fracturing is then predicted.

#### Assumptions

The assumptions used are discussed in an earlier section. Using the Mont Terri data for the Opalinus Clay in Germany is questionable as the site is at least 100 km from any likely radioactive waste storage site in Germany. Furthermore, the Mont Terri Rock Laboratory is in a Late Miocene thrust anticline,^118^ so is in a very different structural and tectonic setting than the likely radioactive waste storage sites in Germany, which must be no closer than 1 km from a fault that has been active since the end of the Eocene. Any site for radioactive waste storage is therefore likely to be outside the region strongly influenced by the Alpine Orogenic Belt. These data may therefore not be representative of the mechanical properties of the Opalinus Clay in Germany.

#### Results of the modeling

The modeling (Figure 8) predicts that fracturing is not likely to occur in intact Opalinus Clay at the maximum burial depth of 2 km, assuming no overpressure or applied tectonic stress. If the Opalinus Clay was cohesional (as opposed to a cohesionless soil; e.g.,^119^), 400 m exhumation would cause the stresses required for generating shear fractures. Note that the differential effective stresses (σ′_Diff_) decrease slightly during exhumation (from 17.93 MPa at 2 km to 17.53 MPa at 1.6 km) because the effect of k_o_ is partly counteracted by thermal cooling, which reduces σ_H_. The model also predicts that exhumation will generate shear fractures rather than joints, partly because of the low cohesion and low coefficient of internal friction of the Opalinus Clay. This suggests that the assumption that exhumation will tend to create joints in the Opalinus Clay may be wrong, especially in the initial stages of exhumation.

The suggestion that faults could develop in the Opalinus Clay, if sufficient exhumation has occurred, is significant because it is a legal requirement that any site chosen for the underground storage of radioactive waste must be more than 1 km from any fault that has been active since the end of the Eocene. We note, however, that faults in the Opalinus Clay developed during exhumation are likely to have small displacements (sub-seismic resolution) and may be sealing.

#### Validity and how modeling of the Opalinus Clay can be improved

We note that Pollard and Aydin^120^ state that, although Mohr diagrams are useful for representing homogeneous stress fields, they do not represent the heterogeneous stress fields associated with joints. We suggest, however, that the modeling presented here is useful because it indicates that faults rather than joints may develop in the Opalinus Clay, at least during the initial stages of exhumation. The starting assumption that exhumation would create joints may therefore be wrong. It is also useful because it highlights a potential issue with the storage of radioactive waste in the Opalinus Clay in areas that have undergone sufficient exhumation to create faults.

The model can be improved by using mechanical properties that are more suitable for potential radioactive waste storage sites in Germany. It also needs to be ground-truthed by the careful analysis of structures in the Opalinus Clay to test what structures have developed during exhumation.

## Discussion and limitations

### Uses of the approach

Here we discuss how Mohr diagrams can foster understanding of fractures in the sub-surfaces to facilitate more sustainable energy production. The ultimate aim may be to develop a model of fractures and fluid flow in the subsurface, such as a discrete fracture network model. The data required for such a model will rarely be available at an early stage of a project, such as before an exploration well has been drilled. The scientist will typically still need to make predictions about the sub-surface, often to convince managers, financial backers and government that it is worth drilling an exploration well. Mohr diagrams provide a simple method, based on a series of overt assumptions, that can help make and justify predictions about fractures in the sub-surface.

Mohr diagrams also have uses beyond the exploration stage, such as deciding which input parameters to use in more sophisticated modeling techniques, and whether the assumptions used in those models are realistic (see “problems with the input data and assumptions”).

The approach can be adapted for solving the same or other problems in other regions. We suggest that the following three points are important in such adaptation. First, any problem is stated as a simple, testable question, such as “how are the different rocks in a potential reservoir likely to respond to thermal or hydraulic stimulation?” Second, a range of appropriate input parameters need to be selected. We had problems deciding what values are appropriate (see next section), but attempted to solve this using reasonable *ranges* of values. Third, while predictions from the modeling should not be treated with high levels of confidence, it is important not to dismiss unexpected results. Such unexpected results challenge assumptions and can lead to greater understanding.

### Problems with the input data and assumptions

Here we discuss problems with the data and assumptions used to create Mohr diagrams, and how Mohr diagrams may be used to identify such problems in more sophisticated models.

Any assessment of failure in the sub-surface relies on good and appropriate estimation of the *in-situ* rock properties. The most difficult part of the modeling presented in this article was finding consistent and reliable input parameters from the literature. For example, Reeher et al.^102^ present a value for the cohesion of quartzite that is higher than the values we selected to be reasonable end-member values for a quartzite (Table 1). Figure 6 shows the importance of rock properties and how the assumed rock properties may be incorrect, with indications that the lowest-case value of Poisson’s ratio being too low. Testing samples and triaxial test results have major problems, both in terms of sampling and reproducing sub-surface stress conditions.^79^ Using Mohr diagrams may help in deciding which input parameters are realistic.

An important use of Mohr diagrams comes from differences between geological data and the model results, which can highlight problems in the assumptions used in the modeling. The assumption of zero horizontal strain (the *uniaxial strain* condition) is also likely to be incorrect as deformation occurs, with horizontal extension or contraction proving the assumption is invalid.^83^ Such problems are limitations to Mohr diagrams and need to be understood and considered when using the approach. We suggest, however, that determining the causes of inconsistencies between geological data and models can provide useful insights into deformation, including about the mechanical properties of the rock and about the boundary conditions during deformation.

These problems associated with both choices of input parameters and modeling assumptions associated with Mohr diagrams tend to be amplified in more sophisticated models. A range of more sophisticated numerical techniques exist for modeling deformation, including distinct element,^121^ discrete element^122^ and thermo-elastic^123^ methods. Such models also make a series of assumptions about the stresses, boundary conditions, rock properties and the way rocks fracture. Furthermore, they often require more input parameters than do Mohr diagrams. For example, discrete fracture network models require such poorly-constrained parameters as fracture apertures and connectivity, with the assumptions made often being buried within the “black-box” software.^124^ Mohr diagrams can be used as a simple method for testing the sensitivities of some of the input parameters used in more sophisticated modeling techniques, and can be used to test the validity of some results from such models.

### Accuracy and precision, and the spread of input parameters and results

Mohr diagrams are a relatively simple graphical technique to predict relationships between stresses, fluid pressure, rock properties and failure. We suggest the following benefits of using Mohr diagrams, particularly when limited data are available.

• The relatively few input parameters used in Mohr diagrams suggests they may be less prone to errors than more sophisticated modeling techniques. This is especially true when input parameters are poorly-constrained, such as at the pre-drill stage of assessing potential reservoir rocks. Results are likely to become increasingly unreliable as the number of poorly-constrained parameters used increases.
• While Mohr diagrams can be constructed quickly and simply using a pencil and paper, or a spreadsheet, more sophisticated modeling techniques typically require powerful (and often expensive) computer programs and take many hours to construct and process. Mohr diagrams can be run more efficiently and the effects of changing individual input parameters can be readily determined. This can be particularly helpful in identifying problems in the assumptions and input parameters used.
• While more sophisticated techniques, such as discrete fracture network modeling, may give precise results, they are likely to have low accuracy if the input parameters are poorly-constrained. We suggest that using Mohr diagrams may give higher accuracy but probably lower precision. That low precision may be advantageous because it shows a range of possible outcomes. The efficiency of Mohr diagrams means that it can be quicker and easier to model spreads of behavior and the effects of changing individual parameters, which helps evaluate end-member scenarios. Predicting the spreads of *possible* behaviors helps prediction of what is *likely* to occur. Such an approach is particularly helpful in predicting deformation in the sub-surface when limited data are available, such as predicting, at the pre-drill stage, the likely effects of thermal or hydraulic stimulation on reservoir rocks. Determining spreads of possible behaviors is compatible with Monte Carlo testing of potential reservoirs, as used in the petroleum^125^^,^^126^^,^^127^ and geothermal^128^ industries.

Three general points about the use of this approach. Firstly, the main problem we had was finding sensible input parameters. This is partly because the examples we discuss are at early stages of project development, with very limited site-specific information available. Secondly, we suggest that the method is most useful at such early stages because the method lends itself well to using spreads of values for each of the input parameters. Our results have led us to question whether the input parameters are appropriate. Thirdly, this means that it is more important to test reasonable spreads of values for the input parameters (e.g., Poisson’s ratio, rock density) than to attempt to find precise (but probably highly inaccurate) values.

### Similarity to section balancing and restoration

Mohr diagrams are a simple graphical method that can be used to test what is likely to occur in the sub-surface, and can give useful information about the development of structures. They can help test the validity of assumptions, such as those made about the boundary conditions of deformation used in the base-case models used here. They share much in common with techniques to balance and restore cross-sections.^129^^,^^130^^,^^131^^,^^132^^,^^133^ Balancing and restoration techniques are graphical methods that enable cross-sections drawn from field or seismic data to be tested and validated.^134^ They help identify problems in cross-sections, such as when a cross-section cannot be balanced or restored. Mohr diagrams and section balancing and restoration techniques are therefore helpful for testing hypotheses and identifying misconceptions.

### Conclusions

Mohr diagrams are a simple graphical method that can be used to make predictions about the subsurface and to test hypotheses, including about the development of fractures. Such uses are illustrated by attempting to answer two questions: (1) how may metasedimentary rocks in the subsurface, which are potential geothermal reservoir rocks, respond to thermal stimulation, and; (2) will joints develop in a clay during exhumation? The results of the modeling to solve these questions are.

(1) It is predicted that quartzites will respond more to thermal stimulation (changes in temperature) than will slates. This is because quartzites generally have lower geostatic stress ratios and higher Young’s moduli and coefficients of thermal expansion than slates. Also, thermal stimulation would tend to reactivate cohesionless fractures in shear mode rather than extensional mode, but would tend to produce new extensional or hybrid fractures. These results need to be treated with caution. For example, the magnitudes and orientations of horizontal stresses are unknown, and there may not be a simple relationship between the cooling of the rocks and the temperature of the injected fluid.
(2) Modeling of the Opalinus Clay predicts shear fractures rather than joints would tend to develop during exhumation. Joint formation would need cohesional behavior and high overpressure. Joints may also develop under low differential stresses, for example if a tectonic compressional stress is applied or if the Opalinus Clay had not been buried as far the 2 km maximum burial depth modeled here. It is possible, however, that using Mohr diagrams is inappropriate for analyzing joint development,^120^ especially because the Opalinus Clay may show ductile rather than brittle deformation. Mohr diagrams have indicated rock behavior that was not predicted before the modeling.

While some of the results presented here indicate weaknesses in the approach, they illustrate a key benefit, which is that Mohr diagrams can be used to test hypotheses and may highlight misconceptions. Those misconceptions are often in the form of the starting assumptions used in the modeling. As such, Mohr diagrams are similar to methods for section balancing and restoration. Both approaches are simple graphical methods that may not *prove* a model is correct, but are useful tests of whether a model *may* be correct. The simplicity of creating Mohr diagrams, including the use of spreadsheets, means that it is straightforward to model the spreads of input parameters individually or together. This is particularly useful at the exploration stage, when predictions may need to be made about ranges of possible behavior in the subsurface. This includes predicting how potential geothermal reservoir rocks are likely to respond to thermal or hydraulic stimulation, and whether or not those reservoir rocks may be jointed.

## STAR★Methods

### Key resources table

**Table:** 

REAGENT or RESOURCE	SOURCE	IDENTIFIER
**Deposited data**		

Rock mechanical properties	Public domain data	References cited

**Software and algorithms**		

Microsoft Excel	Microsoft	https://www.microsoft.com/en-us/microsoft-365/excel

### Resources availability

#### Lead contact

D.C.P. Peacock is the lead contact. For information, use hermangedge@gmail.com.

#### Materials availability

This study did not generate any new materials.

#### Data and code availability

All data and Mohr diagram models reported in this paper will be shared by the lead contact upon request.

### Method details

Mohr diagrams are used with public-domain data to model how metasedimentary rocks may respond to thermal stimulation, and whether fractures may develop in a clay sequence during exhumation.
